# 2-[(4-Meth­oxy-2-nitro­phen­yl)imino­meth­yl]phenol

**DOI:** 10.1107/S1600536812031212

**Published:** 2012-07-14

**Authors:** Aliakbar Dehno Khalaji, Mahsa Nikookar, Karla Fejfarová, Michal Dušek

**Affiliations:** a Department of Chemistry, Faculty of Science, Golestan University, Gorgan, Iran; b Institute of Physics ASCR, v.v.i., Na Slovance 2, 182 21 Prague 8, Czech Republic

## Abstract

The crystal structure of the title compound, C_14_H_12_N_2_O_4_, contains four crystallographically independent mol­ecules in the asymmetric unit. All the mol­ecules have similar conformations; the dihedral angles between the aromatic rings are 33.1 (1), 33.76 (9), 31.41 (9) and 32.56 (10)°. Intra­molecular O—H⋯N hydrogen bonds form *S*(6) ring motifs in each molecule. In the crystal, there are two pairs of pseudo-inversion-related mol­ecules. Along the *c* axis, mol­ecules are stacked with π–π inter­actions between the 2-hy­droxy­phenyl and 4-meth­oxy-2-nitro­phenyl rings [centroid–centroid distances = 3.5441 (12)–3.7698 (12) Å].

## Related literature

For related structures, see: Akkurt *et al.* (2008 ▶); Fejfarová *et al.* (2010 ▶); Fun *et al.* (2009 ▶); Kargar *et al.* (2012 ▶); Keleşoğlu *et al.* (2009 ▶); Khalaji *et al.* (2007 ▶); Özek *et al.* (2009 ▶); Tanak *et al.* (2009 ▶). For the extinction correction, see: Becker & Coppens (1974 ▶).
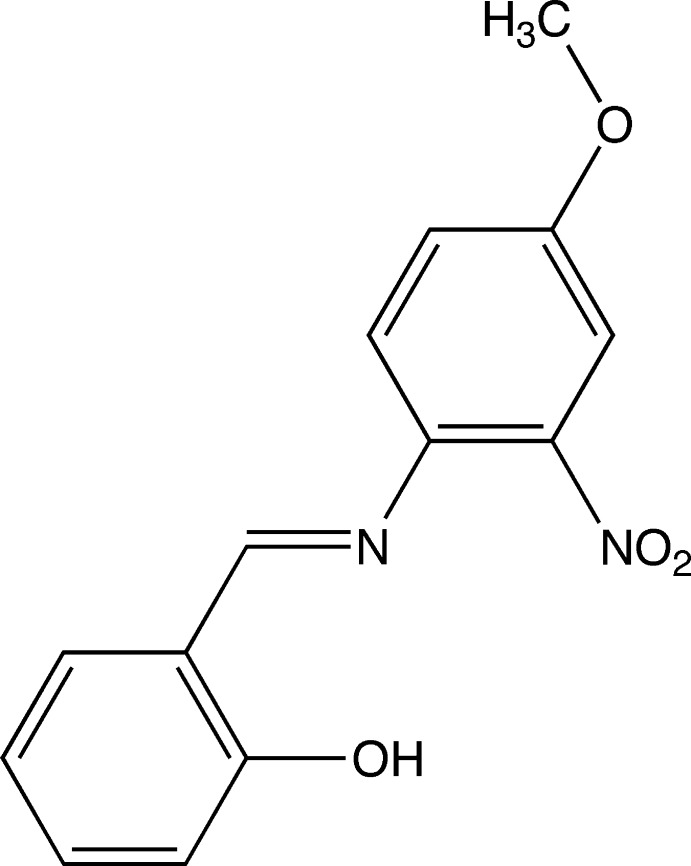


## Experimental

### 

#### Crystal data

C_14_H_12_N_2_O_4_
*M* *_r_* = 272.3
Monoclinic, 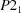

*a* = 16.8655 (2) Å
*b* = 21.0838 (5) Å
*c* = 7.0741 (5) Å
β = 90.817 (2)°
*V* = 2515.22 (19) Å^3^
*Z* = 8
Cu *K*α radiation
μ = 0.9 mm^−1^
*T* = 120 K
0.25 × 0.20 × 0.16 mm

#### Data collection

Agilent Xcalibur diffractometer with an Atlas (Gemini ultra Cu) detector
Absorption correction: multi-scan (*CrysAlis PRO*; Agilent, 2012 ▶) *T*_min_ = 0.851, *T*_max_ = 1
19454 measured reflections
7375 independent reflections
6684 reflections with *I* > 3σ(*I*)
*R*_int_ = 0.024

#### Refinement

*R*[*F* ^2^ > 3σ(*F* ^2^)] = 0.033
*wR*(*F* ^2^) = 0.087
*S* = 1.38
7375 reflections
735 parameters
H atoms treated by a mixture of independent and constrained refinement
Δρ_max_ = 0.15 e Å^−3^
Δρ_min_ = −0.12 e Å^−3^
Absolute structure: Flack (1983 ▶), 2778 Friedel pairs
Flack parameter: −0.06 (15)

### 

Data collection: *CrysAlis PRO* (Agilent, 2012 ▶); cell refinement: *CrysAlis PRO*; data reduction: *CrysAlis PRO*; program(s) used to solve structure: *SIR2002* (Burla *et al.*, 2003 ▶); program(s) used to refine structure: *JANA2006* (Petříček *et al.*, 2006 ▶); molecular graphics: *DIAMOND* (Brandenburg & Putz, 2005 ▶); software used to prepare material for publication: *JANA2006*.

## Supplementary Material

Crystal structure: contains datablock(s) global, I. DOI: 10.1107/S1600536812031212/fk2064sup1.cif

Structure factors: contains datablock(s) I. DOI: 10.1107/S1600536812031212/fk2064Isup2.hkl

Supplementary material file. DOI: 10.1107/S1600536812031212/fk2064Isup3.cml

Additional supplementary materials:  crystallographic information; 3D view; checkCIF report

## Figures and Tables

**Table 1:** Hydrogen-bond geometry (Å, °)

*D*—H⋯*A*	*D*—H	H⋯*A*	*D*⋯*A*	*D*—H⋯*A*
O104—H104*o*⋯N102	0.86 (3)	1.81 (3)	2.593 (2)	150 (3)
O204—H204*o*⋯N202	0.88 (3)	1.81 (3)	2.599 (2)	148 (3)
O304—H304*o*⋯N302	0.92 (3)	1.76 (3)	2.593 (2)	148 (3)
O404—H404*o*⋯N402	0.90 (3)	1.79 (3)	2.598 (2)	148 (3)

**Fig. 1.**
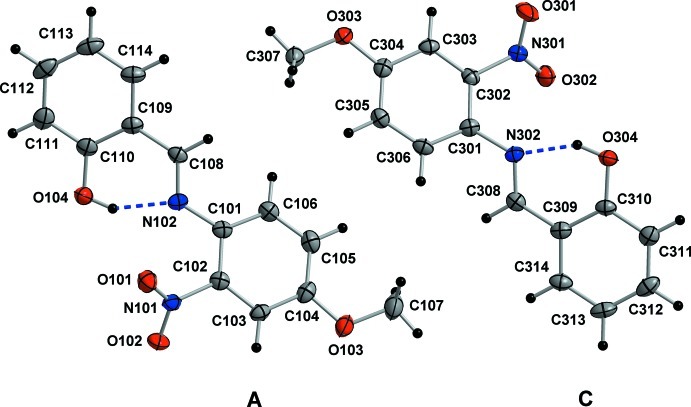
Molecules A and C of the title compound. Displacement ellipsoids are shown at the 50% probability level. Hydrogen bonds are drawn as dashed lines.

**Fig. 2.**
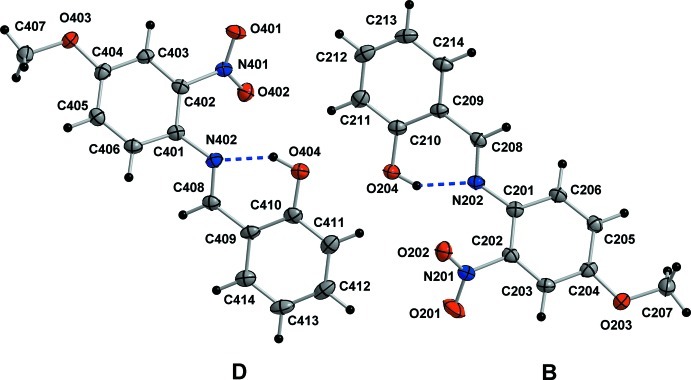
Molecules B and D of the title compound. Displacement ellipsoids are shown at the 50% probability level. Hydrogen bonds are drawn as dashed lines.

**Fig. 3.**
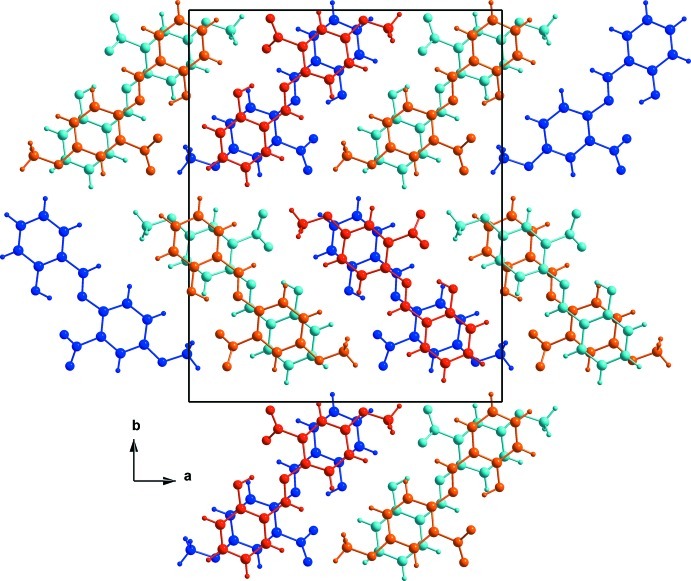
Packing of molecules viewed along c-axis. [red: A, blue: B, orange: C, cyan: D]

## supplementary crystallographic information

### Comment

The present work is part of a structural study of Schiff bases (Khalaji *et al.*, 2007; Fejfarová *et al.*, 2010). The crystal structure contains four crystallographically independent molecules (A–D) in the asymmetric unit (Fig. 1 and 2). All the molecules have similar conformations, the dihedral angles between the two aromatic rings are 33.1 (1)°, 33.76 (9)°, 31.41 (9)°, and 32.56 (10)°. The azomethine functional groups are co-planar with the adjacent benzene rings. The dihedral angles between the planes of the benzene ring and the plane defined by C(aromatic)—C=N atoms are 2.8 (2)°, 1.50 (18)°, 3.19 (19)°, and 2.7 (2)°. The methoxy groups are almost coplanar with the adjacent benzene rings as can be seen from the torsion angles Cx03—Cx04—Ox03—Cx07 (x = 1–4 for molecules A–D) of -174.57 (19)°, -176.30 (17)°, 174.10 (17)°, and 176.27 (17)°, respectively. The average of C═N and C—N bond lengths of 1.286 and 1.407 Å agree well with the corresponding distances in other Schiff bases (Akkurt *et al.*, 2008; Fun *et al.*, 2009; Kargar *et al.*, 2012; Keleşoğlu *et al.*, 2009; Özek *et al.*, 2009; Tanak *et al.*, 2009.

In the crystal, there are two pairs (A+C, B+D) of pseudo inversion-related molecules. The phenol H atoms form strong intramolecular Ox04—H···Nx02 hydrogen bonds with the imine N atoms, generating S(6) ring motifs. Along the *c* axis, the molecules are stacked with π-π interactions between the 2-hydroxyphenyl and 4-methoxy-2-nitrophenyl rings of A+B and C+D pairs [centroid-centroid distances in the range of 3.5441 (12)Å – 3.7698 (12) Å] (Figure 3).

### Experimental

To a stirred solution of the salicylaldehyde (0.2 mmol, in 5 ml of methanol) was added 4-methoxy-2-nitroaniline (0.2 mmol) in 10 ml of methanol and the mixture was stirred for 1 h in air at 323 K and was then left at room temperature for several days without disturbance yielding suitable crystals of the title compound that subsequently were filtered off and washed with Et_2_O. Yield: 88%. Yellow crystals. Anal. Calc. for C_14_H_12_N_2_O_4_: C, 61.76; H, 4.44; N, 10.29%. Found: C, 61.70; H, 4.51; N, 10.38%.

### Refinement

Hydrogen atoms attached to carbons were kept in ideal positions with C–H distance 0.96 Å during the refinement. The methyl H atoms were allowed to rotate freely about the adjacent C—O bonds. The hydroxyl hydrogen atoms were found in difference Fourier maps and their coordinates were refined freely. The isotropic atomic displacement parameters of hydrogen atoms were set to 1.5*U*_eq_ (methyl and hydroxyl groups) or 1.2*U*_eq_ of the parent atom. Reflections -1 19 - 5 and 1 18 - 5 were omitted in last cycles of refinement as outliers.

### Crystal data

**Table:**

C_14_H_12_N_2_O_4_	*F*(000) = 1136
*M**_r_* = 272.3	*D*_x_ = 1.438 Mg m^−^^3^
Monoclinic, *P*2_1_	Cu *K*α radiation, λ = 1.5418 Å
Hall symbol: P 2yb	Cell parameters from 12919 reflections
*a* = 16.8655 (2) Å	θ = 3.4–67.6°
*b* = 21.0838 (5) Å	µ = 0.9 mm^−^^1^
*c* = 7.0741 (5) Å	*T* = 120 K
β = 90.817 (2)°	Prism, yellow
*V* = 2515.22 (19) Å^3^	0.25 × 0.20 × 0.16 mm
*Z* = 8	

### Data collection

**Table:**

Agilent Xcalibur diffractometer with an Atlas (Gemini ultra Cu) detector	7375 independent reflections
Radiation source: Enhance Ultra (Cu) X-ray Source	6684 reflections with *I* > 3σ(*I*)
Mirror monochromator	*R*_int_ = 0.024
Detector resolution: 10.3784 pixels mm^-1^	θ_max_ = 67.1°, θ_min_ = 3.4°
Rotation method data acquisition using ω scans	*h* = −20→20
Absorption correction: multi-scan (*CrysAlis PRO*; Agilent, 2012)	*k* = −25→19
*T*_min_ = 0.851, *T*_max_ = 1	*l* = −8→8
19454 measured reflections	

### Refinement

**Table:**

Refinement on *F*^2^	Weighting scheme based on measured s.u.'s *w* = 1/(σ^2^(*I*) + 0.0016*I*^2^)
*R*[*F*^2^ > 2σ(*F*^2^)] = 0.033	(Δ/σ)_max_ = 0.040
*wR*(*F*^2^) = 0.087	Δρ_max_ = 0.15 e Å^−^^3^
*S* = 1.38	Δρ_min_ = −0.12 e Å^−^^3^
7375 reflections	Extinction correction: Becker & Coppens (1974), B-C type 1 Lorentzian isotropic
735 parameters	Extinction coefficient: 23 (3)
0 restraints	Absolute structure: Flack (1983), 2778 Friedel pairs
181 constraints	Flack parameter: −0.06 (15)
H atoms treated by a mixture of independent and constrained refinement	

### Special details

**Table:**

Experimental. CrysAlis PRO (Agilent, 2012) Empirical absorption correction using spherical harmonics, implemented in SCALE3 ABSPACK scaling algorithm.
Refinement. The refinement was carried out against all reflections. The conventional *R*-factor is always based on *F*. The goodness of fit as well as the weighted *R*-factor are based on *F* and *F*^2^ for refinement carried out on *F* and *F*^2^, respectively. The threshold expression is used only for calculating *R*-factors *etc*. and it is not relevant to the choice of reflections for refinement.The program used for refinement, Jana2006, uses the weighting scheme based on the experimental expectations, see _refine_ls_weighting_details, that does not force *S* to be one. Therefore the values of *S* are usually larger than the ones from the *SHELX* program.

### Fractional atomic coordinates and isotropic or equivalent isotropic displacement parameters (Å^2^)

**Table:**

	*x*	*y*	*z*	*U*_iso_*/*U*_eq_	
O101	0.75197 (9)	0.40161 (8)	0.2684 (2)	0.0325 (5)	
O102	0.73997 (10)	0.47881 (8)	0.0692 (3)	0.0418 (5)	
O103	0.44321 (9)	0.47415 (8)	0.0226 (2)	0.0353 (5)	
O104	0.84363 (9)	0.28817 (7)	−0.0391 (2)	0.0295 (4)	
O201	0.62031 (12)	0.09153 (10)	0.5791 (4)	0.0695 (8)	
O202	0.60210 (9)	0.17182 (8)	0.7624 (2)	0.0325 (5)	
O203	0.91500 (8)	0.10142 (7)	0.5481 (2)	0.0290 (4)	
O204	0.51076 (9)	0.28366 (7)	0.4491 (2)	0.0267 (4)	
O301	0.12187 (11)	0.09018 (9)	0.1065 (3)	0.0560 (7)	
O302	0.09907 (9)	0.17300 (7)	−0.0624 (2)	0.0313 (5)	
O303	0.41587 (8)	0.10577 (7)	0.1458 (2)	0.0283 (4)	
O304	0.00811 (9)	0.28192 (7)	0.2504 (2)	0.0286 (4)	
O401	0.23694 (9)	0.48015 (8)	0.6054 (3)	0.0401 (5)	
O402	0.25409 (8)	0.39560 (7)	0.4393 (2)	0.0303 (4)	
O403	−0.05477 (8)	0.46909 (7)	0.6735 (2)	0.0283 (4)	
O404	0.34982 (9)	0.28420 (7)	0.7316 (2)	0.0295 (4)	
N101	0.71506 (10)	0.43047 (8)	0.1454 (2)	0.0252 (5)	
N102	0.69505 (10)	0.30308 (8)	0.0389 (2)	0.0231 (5)	
N201	0.64206 (10)	0.14142 (9)	0.6521 (2)	0.0281 (5)	
N202	0.65943 (9)	0.26907 (8)	0.5403 (2)	0.0212 (5)	
N301	0.14073 (10)	0.14177 (8)	0.0458 (2)	0.0263 (5)	
N302	0.15641 (9)	0.26977 (8)	0.1656 (2)	0.0219 (5)	
N401	0.21568 (10)	0.42781 (8)	0.5491 (2)	0.0235 (5)	
N402	0.19913 (10)	0.29979 (8)	0.6657 (2)	0.0219 (5)	
C101	0.62753 (12)	0.34198 (10)	0.0459 (2)	0.0226 (6)	
C102	0.63683 (12)	0.40674 (10)	0.0865 (2)	0.0228 (5)	
C103	0.57583 (12)	0.45023 (10)	0.0748 (3)	0.0250 (6)	
C104	0.50037 (12)	0.42838 (11)	0.0271 (3)	0.0269 (6)	
C105	0.48848 (12)	0.36469 (11)	−0.0147 (3)	0.0283 (6)	
C106	0.55178 (12)	0.32283 (11)	−0.0075 (3)	0.0256 (6)	
C107	0.36311 (14)	0.45354 (14)	−0.0091 (4)	0.0463 (8)	
C108	0.69080 (12)	0.24352 (10)	0.0766 (3)	0.0231 (6)	
C109	0.75967 (12)	0.20240 (10)	0.0614 (3)	0.0233 (6)	
C110	0.83385 (12)	0.22653 (10)	0.0064 (3)	0.0249 (6)	
C111	0.89955 (14)	0.18668 (11)	0.0031 (3)	0.0315 (7)	
C112	0.89183 (15)	0.12361 (11)	0.0522 (3)	0.0349 (7)	
C113	0.81873 (15)	0.09888 (11)	0.1029 (3)	0.0345 (7)	
C114	0.75348 (14)	0.13811 (10)	0.1074 (3)	0.0288 (6)	
C201	0.72755 (11)	0.23065 (10)	0.5557 (2)	0.0211 (5)	
C202	0.71981 (11)	0.16640 (10)	0.5997 (2)	0.0220 (5)	
C203	0.78191 (12)	0.12391 (10)	0.5954 (3)	0.0231 (5)	
C204	0.85700 (12)	0.14623 (10)	0.5498 (3)	0.0235 (5)	
C205	0.86774 (12)	0.21004 (10)	0.5059 (3)	0.0243 (6)	
C206	0.80306 (12)	0.25102 (10)	0.5070 (3)	0.0233 (6)	
C207	0.99420 (13)	0.12213 (11)	0.5134 (3)	0.0344 (7)	
C208	0.66305 (12)	0.32867 (9)	0.5764 (2)	0.0212 (5)	
C209	0.59460 (12)	0.36982 (9)	0.5515 (2)	0.0215 (5)	
C210	0.52078 (12)	0.34580 (10)	0.4906 (3)	0.0225 (5)	
C211	0.45560 (13)	0.38634 (10)	0.4734 (3)	0.0273 (6)	
C212	0.46387 (14)	0.44984 (11)	0.5144 (3)	0.0294 (6)	
C213	0.53661 (13)	0.47458 (10)	0.5748 (3)	0.0292 (6)	
C214	0.60111 (13)	0.43468 (10)	0.5936 (3)	0.0258 (6)	
C301	0.22495 (12)	0.23216 (10)	0.1496 (2)	0.0222 (5)	
C302	0.21824 (11)	0.16775 (10)	0.1029 (2)	0.0219 (5)	
C303	0.28126 (12)	0.12637 (10)	0.1048 (3)	0.0235 (6)	
C304	0.35633 (11)	0.14957 (10)	0.1511 (2)	0.0227 (5)	
C305	0.36609 (12)	0.21300 (10)	0.1999 (3)	0.0248 (6)	
C306	0.30082 (12)	0.25293 (10)	0.2010 (3)	0.0246 (6)	
C307	0.49501 (13)	0.12832 (11)	0.1742 (3)	0.0331 (7)	
C308	0.15887 (12)	0.32986 (10)	0.1339 (2)	0.0225 (6)	
C309	0.09020 (12)	0.36993 (10)	0.1613 (3)	0.0230 (6)	
C310	0.01678 (12)	0.34448 (10)	0.2162 (3)	0.0231 (6)	
C311	−0.04890 (13)	0.38389 (10)	0.2325 (3)	0.0275 (6)	
C312	−0.04221 (14)	0.44799 (11)	0.2012 (3)	0.0315 (6)	
C313	0.03044 (14)	0.47442 (11)	0.1530 (3)	0.0321 (6)	
C314	0.09536 (14)	0.43544 (10)	0.1301 (3)	0.0285 (6)	
C401	0.13152 (12)	0.33874 (10)	0.6584 (2)	0.0214 (5)	
C402	0.13938 (11)	0.40338 (10)	0.6143 (2)	0.0205 (5)	
C403	0.07786 (11)	0.44609 (10)	0.6232 (3)	0.0221 (5)	
C404	0.00308 (12)	0.42425 (10)	0.6733 (3)	0.0238 (6)	
C405	−0.00726 (12)	0.36086 (10)	0.7203 (3)	0.0242 (6)	
C406	0.05651 (12)	0.31940 (10)	0.7147 (3)	0.0236 (6)	
C407	−0.13379 (13)	0.44822 (11)	0.7128 (3)	0.0328 (7)	
C408	0.19402 (12)	0.24032 (10)	0.6266 (3)	0.0225 (6)	
C409	0.26229 (12)	0.19855 (9)	0.6426 (3)	0.0227 (6)	
C410	0.33761 (12)	0.22219 (10)	0.6938 (3)	0.0246 (6)	
C411	0.40245 (13)	0.18123 (11)	0.7024 (3)	0.0284 (6)	
C412	0.39276 (14)	0.11775 (11)	0.6651 (3)	0.0319 (6)	
C413	0.31855 (14)	0.09349 (11)	0.6156 (3)	0.0328 (7)	
C414	0.25409 (13)	0.13381 (10)	0.6036 (3)	0.0275 (6)	
H103	0.585237	0.494417	0.098954	0.03*	
H105	0.436446	0.349691	−0.048573	0.0339*	
H106	0.542941	0.279209	−0.040526	0.0308*	
H107a	0.349617	0.422274	0.083706	0.0694*	
H107b	0.358146	0.435414	−0.133174	0.0694*	
H107c	0.32794	0.489144	0.001004	0.0694*	
H108	0.641174	0.225844	0.115451	0.0277*	
H111	0.950222	0.203014	−0.033335	0.0378*	
H112	0.93754	0.096486	0.051263	0.0418*	
H113	0.813774	0.054795	0.134483	0.0414*	
H114	0.703024	0.121068	0.14264	0.0346*	
H203	0.773585	0.079867	0.623359	0.0277*	
H205	0.91944	0.225647	0.475056	0.0291*	
H206	0.810756	0.294662	0.473175	0.0279*	
H207a	1.028774	0.086013	0.508641	0.0517*	
H207b	0.995611	0.144328	0.395029	0.0517*	
H207c	1.011379	0.149947	0.613318	0.0517*	
H208	0.712229	0.3465	0.621055	0.0254*	
H211	0.405049	0.369938	0.432901	0.0327*	
H212	0.418902	0.477488	0.501307	0.0353*	
H213	0.541765	0.518939	0.60294	0.0351*	
H214	0.651151	0.451524	0.63617	0.031*	
H303	0.273638	0.082359	0.074844	0.0282*	
H305	0.417752	0.229055	0.232625	0.0298*	
H306	0.308043	0.296346	0.238293	0.0296*	
H307a	0.531577	0.093827	0.159668	0.0497*	
H307b	0.50616	0.160643	0.082765	0.0497*	
H307c	0.500475	0.145641	0.299198	0.0497*	
H308	0.207323	0.348547	0.091177	0.027*	
H311	−0.099248	0.3662	0.265864	0.033*	
H312	−0.087953	0.474743	0.212543	0.0378*	
H313	0.035335	0.519407	0.135897	0.0386*	
H314	0.144888	0.453517	0.092136	0.0341*	
H403	0.086317	0.490123	0.595399	0.0265*	
H405	−0.058516	0.345747	0.756774	0.0291*	
H406	0.048689	0.275975	0.750693	0.0283*	
H407a	−0.169637	0.483457	0.702199	0.0492*	
H407b	−0.149166	0.415957	0.623804	0.0492*	
H407c	−0.13547	0.431289	0.838642	0.0492*	
H408	0.143904	0.223143	0.585864	0.027*	
H411	0.454087	0.19746	0.73458	0.0341*	
H412	0.437627	0.089816	0.673308	0.0382*	
H413	0.312213	0.049057	0.589897	0.0393*	
H414	0.203029	0.117191	0.567973	0.033*	
H104o	0.7988 (18)	0.3068 (15)	−0.021 (4)	0.0442*	
H204o	0.5554 (17)	0.2640 (14)	0.477 (4)	0.04*	
H304o	0.0559 (18)	0.2624 (14)	0.227 (4)	0.0429*	
H404o	0.3030 (18)	0.3046 (15)	0.716 (4)	0.0442*	

### Atomic displacement parameters (Å^2^)

**Table:**

	*U*^11^	*U*^22^	*U*^33^	*U*^12^	*U*^13^	*U*^23^
O101	0.0300 (7)	0.0356 (9)	0.0317 (7)	−0.0009 (7)	−0.0051 (6)	0.0042 (7)
O102	0.0340 (8)	0.0241 (9)	0.0675 (11)	−0.0051 (7)	0.0032 (7)	0.0137 (8)
O103	0.0268 (7)	0.0371 (9)	0.0421 (8)	0.0079 (7)	0.0018 (6)	0.0058 (7)
O104	0.0289 (7)	0.0236 (8)	0.0361 (8)	−0.0032 (6)	0.0050 (6)	−0.0027 (6)
O201	0.0451 (11)	0.0350 (11)	0.129 (2)	−0.0194 (9)	0.0263 (12)	−0.0368 (12)
O202	0.0276 (7)	0.0353 (9)	0.0348 (7)	0.0009 (7)	0.0082 (6)	−0.0014 (7)
O203	0.0239 (7)	0.0248 (8)	0.0383 (8)	0.0033 (6)	0.0005 (5)	−0.0023 (6)
O204	0.0271 (7)	0.0183 (7)	0.0346 (7)	−0.0015 (6)	−0.0037 (6)	−0.0005 (6)
O301	0.0380 (9)	0.0309 (10)	0.0987 (15)	−0.0136 (8)	−0.0127 (9)	0.0267 (10)
O302	0.0289 (7)	0.0313 (9)	0.0336 (7)	−0.0014 (6)	−0.0066 (6)	0.0010 (6)
O303	0.0243 (7)	0.0243 (8)	0.0364 (7)	0.0028 (6)	0.0013 (5)	0.0041 (6)
O304	0.0288 (7)	0.0189 (8)	0.0381 (8)	−0.0035 (6)	0.0052 (6)	−0.0026 (6)
O401	0.0364 (8)	0.0198 (8)	0.0643 (10)	−0.0075 (7)	0.0074 (7)	−0.0083 (8)
O402	0.0279 (7)	0.0298 (8)	0.0335 (7)	0.0019 (6)	0.0063 (6)	−0.0027 (6)
O403	0.0229 (7)	0.0236 (8)	0.0384 (7)	0.0054 (6)	0.0025 (5)	−0.0035 (6)
O404	0.0279 (7)	0.0222 (8)	0.0383 (8)	−0.0007 (6)	−0.0015 (6)	0.0021 (6)
N101	0.0276 (8)	0.0192 (9)	0.0290 (8)	0.0009 (7)	0.0048 (7)	−0.0026 (7)
N102	0.0275 (8)	0.0215 (9)	0.0202 (7)	0.0006 (7)	0.0015 (6)	−0.0008 (6)
N201	0.0258 (9)	0.0199 (9)	0.0388 (9)	−0.0021 (7)	0.0029 (7)	0.0007 (7)
N202	0.0244 (8)	0.0195 (9)	0.0197 (7)	−0.0003 (7)	0.0021 (6)	0.0004 (6)
N301	0.0257 (9)	0.0201 (9)	0.0331 (9)	−0.0017 (7)	0.0006 (7)	0.0004 (7)
N302	0.0252 (8)	0.0196 (9)	0.0210 (7)	0.0006 (7)	−0.0005 (6)	−0.0014 (6)
N401	0.0243 (8)	0.0184 (9)	0.0277 (8)	0.0004 (7)	−0.0003 (6)	0.0027 (7)
N402	0.0249 (8)	0.0189 (9)	0.0220 (7)	0.0020 (7)	0.0015 (6)	0.0023 (6)
C101	0.0274 (10)	0.0222 (11)	0.0181 (8)	−0.0006 (8)	0.0035 (7)	0.0019 (7)
C102	0.0267 (9)	0.0239 (11)	0.0178 (8)	−0.0007 (8)	0.0011 (7)	0.0017 (7)
C103	0.0298 (10)	0.0218 (11)	0.0235 (9)	0.0026 (8)	0.0041 (7)	0.0030 (8)
C104	0.0273 (10)	0.0318 (12)	0.0218 (9)	0.0073 (9)	0.0029 (8)	0.0052 (8)
C105	0.0256 (10)	0.0364 (13)	0.0228 (9)	−0.0030 (9)	−0.0009 (7)	0.0027 (8)
C106	0.0283 (10)	0.0274 (11)	0.0213 (9)	−0.0029 (9)	0.0009 (7)	−0.0005 (8)
C107	0.0274 (12)	0.0528 (17)	0.0588 (15)	0.0084 (11)	0.0010 (10)	0.0112 (13)
C108	0.0266 (10)	0.0225 (11)	0.0200 (8)	−0.0032 (8)	−0.0006 (7)	−0.0017 (8)
C109	0.0320 (10)	0.0212 (11)	0.0168 (8)	0.0001 (8)	−0.0009 (7)	−0.0032 (7)
C110	0.0300 (10)	0.0249 (11)	0.0197 (8)	−0.0015 (8)	−0.0010 (7)	−0.0062 (7)
C111	0.0319 (11)	0.0365 (13)	0.0261 (10)	0.0031 (9)	−0.0018 (8)	−0.0113 (9)
C112	0.0454 (13)	0.0328 (13)	0.0262 (10)	0.0136 (10)	−0.0056 (9)	−0.0087 (9)
C113	0.0542 (14)	0.0233 (11)	0.0258 (10)	0.0059 (10)	−0.0035 (9)	−0.0026 (8)
C114	0.0420 (12)	0.0208 (11)	0.0236 (9)	−0.0014 (9)	−0.0004 (8)	0.0007 (8)
C201	0.0245 (9)	0.0214 (10)	0.0174 (8)	−0.0003 (8)	−0.0002 (7)	−0.0017 (7)
C202	0.0238 (9)	0.0218 (10)	0.0206 (8)	−0.0023 (8)	0.0020 (7)	−0.0034 (7)
C203	0.0288 (10)	0.0173 (10)	0.0231 (9)	−0.0010 (8)	0.0012 (7)	−0.0011 (7)
C204	0.0256 (10)	0.0245 (11)	0.0203 (8)	0.0033 (8)	−0.0017 (7)	−0.0037 (8)
C205	0.0246 (10)	0.0249 (11)	0.0234 (9)	−0.0019 (8)	0.0006 (7)	−0.0009 (8)
C206	0.0264 (10)	0.0216 (11)	0.0219 (9)	−0.0021 (8)	0.0012 (7)	0.0002 (7)
C207	0.0243 (10)	0.0339 (13)	0.0452 (12)	0.0014 (9)	−0.0002 (9)	−0.0069 (10)
C208	0.0242 (9)	0.0210 (10)	0.0185 (8)	−0.0033 (8)	0.0026 (7)	0.0010 (7)
C209	0.0282 (10)	0.0189 (10)	0.0176 (8)	0.0002 (8)	0.0040 (7)	0.0024 (7)
C210	0.0286 (10)	0.0201 (10)	0.0189 (8)	−0.0003 (8)	0.0016 (7)	0.0012 (7)
C211	0.0288 (10)	0.0289 (11)	0.0242 (9)	0.0028 (9)	0.0026 (7)	0.0055 (8)
C212	0.0378 (12)	0.0251 (11)	0.0254 (9)	0.0082 (9)	0.0057 (8)	0.0040 (8)
C213	0.0451 (12)	0.0172 (10)	0.0256 (9)	0.0020 (9)	0.0094 (8)	0.0021 (8)
C214	0.0338 (11)	0.0217 (11)	0.0221 (9)	−0.0031 (8)	0.0030 (8)	0.0002 (8)
C301	0.0274 (10)	0.0213 (10)	0.0179 (8)	0.0007 (8)	0.0019 (7)	0.0006 (7)
C302	0.0237 (9)	0.0218 (10)	0.0202 (8)	−0.0025 (8)	0.0022 (7)	0.0039 (7)
C303	0.0298 (10)	0.0194 (10)	0.0213 (8)	−0.0002 (8)	0.0022 (7)	0.0025 (8)
C304	0.0244 (9)	0.0254 (11)	0.0182 (8)	0.0014 (8)	0.0023 (7)	0.0053 (7)
C305	0.0244 (10)	0.0278 (11)	0.0222 (8)	−0.0024 (9)	0.0001 (7)	0.0002 (8)
C306	0.0300 (10)	0.0218 (11)	0.0221 (9)	−0.0039 (8)	0.0000 (7)	−0.0019 (7)
C307	0.0240 (10)	0.0324 (13)	0.0430 (12)	0.0006 (9)	0.0027 (9)	0.0079 (9)
C308	0.0254 (9)	0.0240 (11)	0.0181 (8)	−0.0030 (8)	−0.0002 (7)	−0.0002 (7)
C309	0.0307 (10)	0.0202 (10)	0.0180 (8)	0.0001 (8)	−0.0019 (7)	−0.0003 (7)
C310	0.0302 (10)	0.0200 (10)	0.0190 (8)	−0.0009 (8)	−0.0016 (7)	−0.0008 (7)
C311	0.0282 (10)	0.0302 (12)	0.0241 (9)	0.0019 (9)	−0.0009 (8)	−0.0037 (8)
C312	0.0408 (12)	0.0298 (12)	0.0238 (9)	0.0124 (10)	−0.0039 (8)	−0.0015 (8)
C313	0.0499 (13)	0.0181 (11)	0.0284 (10)	0.0052 (10)	−0.0004 (9)	0.0019 (8)
C314	0.0380 (11)	0.0229 (11)	0.0244 (9)	−0.0031 (9)	0.0004 (8)	0.0030 (8)
C401	0.0255 (9)	0.0200 (10)	0.0185 (8)	0.0009 (8)	−0.0018 (7)	−0.0011 (7)
C402	0.0249 (9)	0.0194 (10)	0.0171 (8)	−0.0019 (8)	−0.0001 (7)	−0.0014 (7)
C403	0.0261 (10)	0.0178 (10)	0.0224 (8)	0.0019 (8)	−0.0006 (7)	−0.0026 (7)
C404	0.0238 (10)	0.0256 (11)	0.0220 (9)	0.0033 (8)	−0.0005 (7)	−0.0044 (8)
C405	0.0244 (10)	0.0263 (11)	0.0221 (9)	−0.0018 (8)	0.0017 (7)	−0.0005 (8)
C406	0.0284 (10)	0.0212 (10)	0.0211 (9)	−0.0004 (8)	0.0021 (7)	0.0015 (7)
C407	0.0233 (10)	0.0327 (13)	0.0426 (12)	0.0016 (9)	0.0027 (9)	−0.0070 (10)
C408	0.0270 (10)	0.0205 (10)	0.0202 (8)	−0.0013 (8)	0.0039 (7)	0.0028 (7)
C409	0.0317 (11)	0.0188 (10)	0.0178 (8)	0.0032 (8)	0.0032 (7)	0.0044 (7)
C410	0.0300 (10)	0.0232 (11)	0.0207 (8)	0.0007 (8)	0.0053 (7)	0.0052 (7)
C411	0.0298 (11)	0.0308 (12)	0.0246 (9)	0.0065 (9)	0.0046 (8)	0.0052 (8)
C412	0.0396 (12)	0.0303 (12)	0.0259 (10)	0.0147 (10)	0.0088 (8)	0.0063 (9)
C413	0.0497 (13)	0.0208 (11)	0.0280 (10)	0.0071 (10)	0.0081 (9)	−0.0013 (8)
C414	0.0358 (11)	0.0224 (11)	0.0244 (9)	0.0017 (9)	0.0037 (8)	0.0003 (8)

### Geometric parameters (Å, º)

**Table:**

O101—N101	1.225 (2)	C205—C206	1.392 (3)
O102—N101	1.230 (2)	C205—H205	0.96
O103—C104	1.364 (3)	C206—H206	0.96
O103—C107	1.434 (3)	C207—H207a	0.96
O104—C110	1.350 (3)	C207—H207b	0.96
O104—H104o	0.86 (3)	C207—H207c	0.96
O201—N201	1.226 (3)	C208—C209	1.453 (3)
O202—N201	1.220 (2)	C208—H208	0.96
O203—C204	1.360 (2)	C209—C210	1.406 (3)
O203—C207	1.430 (3)	C209—C214	1.403 (3)
O204—C210	1.353 (2)	C210—C211	1.397 (3)
O204—H204o	0.88 (3)	C211—C212	1.376 (3)
O301—N301	1.214 (3)	C211—H211	0.96
O302—N301	1.224 (2)	C212—C213	1.394 (3)
O303—C304	1.365 (2)	C212—H212	0.96
O303—C307	1.428 (3)	C213—C214	1.380 (3)
O304—C310	1.349 (2)	C213—H213	0.96
O304—H304o	0.92 (3)	C214—H214	0.96
O401—N401	1.225 (2)	C301—C302	1.402 (3)
O402—N401	1.224 (2)	C301—C306	1.396 (3)
O403—C404	1.358 (2)	C302—C303	1.375 (3)
O403—C407	1.434 (3)	C303—C304	1.392 (3)
O404—C410	1.350 (3)	C303—H303	0.96
O404—H404o	0.90 (3)	C304—C305	1.390 (3)
N101—C102	1.466 (3)	C305—C306	1.386 (3)
N102—C101	1.405 (3)	C305—H305	0.96
N102—C108	1.286 (3)	C306—H306	0.96
N201—C202	1.466 (3)	C307—H307a	0.96
N202—C201	1.409 (3)	C307—H307b	0.96
N202—C208	1.284 (3)	C307—H307c	0.96
N301—C302	1.469 (3)	C308—C309	1.449 (3)
N302—C301	1.408 (3)	C308—H308	0.96
N302—C308	1.287 (3)	C309—C310	1.409 (3)
N401—C402	1.467 (2)	C309—C314	1.402 (3)
N402—C401	1.406 (3)	C310—C311	1.391 (3)
N402—C408	1.287 (3)	C311—C312	1.374 (3)
C101—C102	1.404 (3)	C311—H311	0.96
C101—C106	1.387 (3)	C312—C313	1.393 (3)
C102—C103	1.380 (3)	C312—H312	0.96
C103—C104	1.390 (3)	C313—C314	1.380 (3)
C103—H103	0.96	C313—H313	0.96
C104—C105	1.389 (3)	C314—H314	0.96
C105—C106	1.386 (3)	C401—C402	1.405 (3)
C105—H105	0.96	C401—C406	1.393 (3)
C106—H106	0.96	C402—C403	1.376 (3)
C107—H107a	0.96	C403—C404	1.393 (3)
C107—H107b	0.96	C403—H403	0.96
C107—H107c	0.96	C404—C405	1.389 (3)
C108—C109	1.455 (3)	C405—C406	1.387 (3)
C108—H108	0.96	C405—H405	0.96
C109—C110	1.410 (3)	C406—H406	0.96
C109—C114	1.398 (3)	C407—H407a	0.96
C110—C111	1.391 (3)	C407—H407b	0.96
C111—C112	1.381 (3)	C407—H407c	0.96
C111—H111	0.96	C408—C409	1.453 (3)
C112—C113	1.390 (3)	C408—H408	0.96
C112—H112	0.96	C409—C410	1.407 (3)
C113—C114	1.377 (3)	C409—C414	1.399 (3)
C113—H113	0.96	C410—C411	1.394 (3)
C114—H114	0.96	C411—C412	1.373 (3)
C201—C202	1.396 (3)	C411—H411	0.96
C201—C206	1.392 (3)	C412—C413	1.392 (3)
C202—C203	1.379 (3)	C412—H412	0.96
C203—C204	1.393 (3)	C413—C414	1.382 (3)
C203—H203	0.96	C413—H413	0.96
C204—C205	1.393 (3)	C414—H414	0.96
			
C104—O103—C107	116.95 (19)	C210—C211—C212	120.0 (2)
C110—O104—H104o	107 (2)	C210—C211—H211	120.01
C204—O203—C207	117.60 (16)	C212—C211—H211	120.01
C210—O204—H204o	107.8 (19)	C211—C212—C213	121.0 (2)
C304—O303—C307	117.21 (16)	C211—C212—H212	119.52
C310—O304—H304o	107.9 (19)	C213—C212—H212	119.52
C404—O403—C407	117.14 (16)	C212—C213—C214	119.4 (2)
C410—O404—H404o	107.8 (19)	C212—C213—H213	120.32
O101—N101—O102	123.36 (18)	C214—C213—H213	120.32
O101—N101—C102	118.60 (16)	C209—C214—C213	120.94 (19)
O102—N101—C102	118.04 (16)	C209—C214—H214	119.53
C101—N102—C108	121.01 (17)	C213—C214—H214	119.53
O201—N201—O202	123.75 (19)	N302—C301—C302	120.06 (17)
O201—N201—C202	117.72 (18)	N302—C301—C306	123.60 (18)
O202—N201—C202	118.52 (17)	C302—C301—C306	115.84 (18)
C201—N202—C208	120.74 (17)	N301—C302—C301	119.65 (17)
O301—N301—O302	123.54 (18)	N301—C302—C303	116.81 (18)
O301—N301—C302	118.27 (17)	C301—C302—C303	123.54 (18)
O302—N301—C302	118.18 (16)	C302—C303—C304	118.67 (19)
C301—N302—C308	120.78 (17)	C302—C303—H303	120.67
O401—N401—O402	123.44 (17)	C304—C303—H303	120.67
O401—N401—C402	117.91 (16)	O303—C304—C303	115.01 (18)
O402—N401—C402	118.64 (16)	O303—C304—C305	124.95 (17)
C401—N402—C408	120.64 (17)	C303—C304—C305	120.03 (19)
N102—C101—C102	119.14 (17)	C304—C305—C306	119.63 (19)
N102—C101—C106	124.35 (18)	C304—C305—H305	120.19
C102—C101—C106	116.02 (18)	C306—C305—H305	120.19
N101—C102—C101	119.18 (17)	C301—C306—C305	122.24 (19)
N101—C102—C103	117.24 (18)	C301—C306—H306	118.88
C101—C102—C103	123.59 (18)	C305—C306—H306	118.88
C102—C103—C104	118.3 (2)	O303—C307—H307a	109.47
C102—C103—H103	120.87	O303—C307—H307b	109.47
C104—C103—H103	120.87	O303—C307—H307c	109.47
O103—C104—C103	114.54 (19)	H307a—C307—H307b	109.47
O103—C104—C105	125.39 (19)	H307a—C307—H307c	109.47
C103—C104—C105	120.1 (2)	H307b—C307—H307c	109.47
C104—C105—C106	119.94 (19)	N302—C308—C309	121.52 (18)
C104—C105—H105	120.03	N302—C308—H308	119.24
C106—C105—H105	120.03	C309—C308—H308	119.24
C101—C106—C105	122.1 (2)	C308—C309—C310	121.47 (18)
C101—C106—H106	118.97	C308—C309—C314	120.14 (19)
C105—C106—H106	118.97	C310—C309—C314	118.38 (19)
O103—C107—H107a	109.47	O304—C310—C309	121.30 (18)
O103—C107—H107b	109.47	O304—C310—C311	118.73 (18)
O103—C107—H107c	109.47	C309—C310—C311	119.96 (19)
H107a—C107—H107b	109.47	C310—C311—C312	120.5 (2)
H107a—C107—H107c	109.47	C310—C311—H311	119.77
H107b—C107—H107c	109.47	C312—C311—H311	119.77
N102—C108—C109	121.33 (18)	C311—C312—C313	120.5 (2)
N102—C108—H108	119.34	C311—C312—H312	119.76
C109—C108—H108	119.34	C313—C312—H312	119.76
C108—C109—C110	121.17 (18)	C312—C313—C314	119.5 (2)
C108—C109—C114	119.90 (19)	C312—C313—H313	120.24
C110—C109—C114	118.90 (19)	C314—C313—H313	120.24
O104—C110—C109	121.68 (18)	C309—C314—C313	121.1 (2)
O104—C110—C111	118.52 (19)	C309—C314—H314	119.43
C109—C110—C111	119.78 (19)	C313—C314—H314	119.43
C110—C111—C112	120.0 (2)	N402—C401—C402	119.72 (17)
C110—C111—H111	120.02	N402—C401—C406	123.94 (18)
C112—C111—H111	120.02	C402—C401—C406	115.88 (18)
C111—C112—C113	120.9 (2)	N401—C402—C401	119.78 (17)
C111—C112—H112	119.57	N401—C402—C403	116.75 (17)
C113—C112—H112	119.57	C401—C402—C403	123.44 (18)
C112—C113—C114	119.5 (2)	C402—C403—C404	118.77 (19)
C112—C113—H113	120.25	C402—C403—H403	120.61
C114—C113—H113	120.25	C404—C403—H403	120.61
C109—C114—C113	121.0 (2)	O403—C404—C403	115.03 (18)
C109—C114—H114	119.52	O403—C404—C405	125.22 (18)
C113—C114—H114	119.52	C403—C404—C405	119.74 (19)
N202—C201—C202	119.76 (17)	C404—C405—C406	119.97 (19)
N202—C201—C206	123.46 (18)	C404—C405—H405	120.02
C202—C201—C206	116.31 (18)	C406—C405—H405	120.02
N201—C202—C201	119.45 (17)	C401—C406—C405	122.12 (19)
N201—C202—C203	117.04 (18)	C401—C406—H406	118.94
C201—C202—C203	123.50 (18)	C405—C406—H406	118.94
C202—C203—C204	118.60 (19)	O403—C407—H407a	109.47
C202—C203—H203	120.7	O403—C407—H407b	109.47
C204—C203—H203	120.7	O403—C407—H407c	109.47
O203—C204—C203	115.07 (18)	H407a—C407—H407b	109.47
O203—C204—C205	124.95 (18)	H407a—C407—H407c	109.47
C203—C204—C205	119.96 (18)	H407b—C407—H407c	109.47
C204—C205—C206	119.59 (18)	N402—C408—C409	121.56 (18)
C204—C205—H205	120.2	N402—C408—H408	119.22
C206—C205—H205	120.2	C409—C408—H408	119.22
C201—C206—C205	121.99 (19)	C408—C409—C410	121.12 (18)
C201—C206—H206	119.01	C408—C409—C414	120.01 (18)
C205—C206—H206	119.01	C410—C409—C414	118.86 (19)
O203—C207—H207a	109.47	O404—C410—C409	121.93 (18)
O203—C207—H207b	109.47	O404—C410—C411	118.29 (18)
O203—C207—H207c	109.47	C409—C410—C411	119.77 (19)
H207a—C207—H207b	109.47	C410—C411—C412	120.3 (2)
H207a—C207—H207c	109.47	C410—C411—H411	119.86
H207b—C207—H207c	109.47	C412—C411—H411	119.86
N202—C208—C209	121.67 (17)	C411—C412—C413	120.7 (2)
N202—C208—H208	119.16	C411—C412—H412	119.65
C209—C208—H208	119.16	C413—C412—H412	119.65
C208—C209—C210	121.37 (18)	C412—C413—C414	119.5 (2)
C208—C209—C214	119.76 (18)	C412—C413—H413	120.22
C210—C209—C214	118.84 (18)	C414—C413—H413	120.23
O204—C210—C209	121.52 (18)	C409—C414—C413	120.8 (2)
O204—C210—C211	118.56 (18)	C409—C414—H414	119.58
C209—C210—C211	119.91 (19)	C413—C414—H414	119.58
			
C103—C104—O103—C107	−174.57 (19)	C303—C304—O303—C307	174.10 (17)
C203—C204—O203—C207	−176.30 (17)	C403—C404—O403—C407	176.27 (17)

### Hydrogen-bond geometry (Å, º)

**Table:**

*D*—H···*A*	*D*—H	H···*A*	*D*···*A*	*D*—H···*A*
O104—H104*o*···N102	0.86 (3)	1.81 (3)	2.593 (2)	150 (3)
O204—H204*o*···N202	0.88 (3)	1.81 (3)	2.599 (2)	148 (3)
O304—H304*o*···N302	0.92 (3)	1.76 (3)	2.593 (2)	148 (3)
O404—H404*o*···N402	0.90 (3)	1.79 (3)	2.598 (2)	148 (3)
